# Electrical Resistivity of Steel Fibre-Reinforced Concrete—Influencing Parameters

**DOI:** 10.3390/ma14123408

**Published:** 2021-06-20

**Authors:** Simon Cleven, Michael Raupach, Thomas Matschei

**Affiliations:** Institute of Building Materials Research, RWTH Aachen University, Schinkelstr. 3, 52062 Aachen, Germany; raupach@ibac.rwth-aachen.de (M.R.); matschei@ibac.rwth-aachen.de (T.M.)

**Keywords:** steel fibre-reinforced concrete, electrical resistivity, fibre content, non-destructive test method

## Abstract

This paper presents a systematic study of the electrical resistivity of different steel fibre-reinforced concretes with fibre contents from 0 kg/m^3^ to 80 kg/m^3^ in order to identify possible effects of interactions among concrete composition and fibre type and content regarding electrical resistivity. Based on a literature review, four parameters, w/c ratio, binder content, ground granulated blast-furnace slag (GGBS) and fineness of cement, which show a significant influence on the electrical resistivity of plain concrete, were identified, and their influence on the electrical resistivity as well as interaction effects were investigated. The results of the experiments highlight that the addition of fibres leads to a significant decrease in electrical resistivity, independent of all additional parameters of the concrete composition. Additionally, it was shown that a higher porosity of the concrete, e.g., due to a higher w/c ratio, also results in a lower electrical resistivity. These results are in agreement with the literature review on plain concrete, while the influence of the concrete composition on the electrical resistivity is weaker with the increase in fibre content. The influence of fibre reinforcement is thus not affected by changes in the concrete composition. In general, a higher fibre dosage leads to a decrease in electrical resistivity, but the impact on the electrical resistivity varies slightly with different types of steel fibres. Based on this study, the potential of determining the fibre content using electrical resistivity measurements could be clearly presented.

## 1. Introduction

As a commonly used composite material for civil construction steel fibre-reinforced concrete (SFRC) combines the positive aspects of both basic materials with respect to its load bearing behaviour (e.g., [1,2,3,4,5,6]). Since the plain concrete has excellent resistance to compressive forces but has almost no ability to withstand tensile stresses, the fibre fraction takes up the remaining tensile forces. With this material, one can produce elements in almost every shape, with sufficient durability and improved mechanical characteristics (e.g., [7,8,9,10,11]). Principal applications of SFRC include industrial floorings, structures in seismic zones or underground construction and applications where structures are exposed to torsion, impact or fatigue (e.g., [12,13,14]). One disadvantage of this type of construction material is the huge influence of fibre content, distribution and orientation on its mechanic properties (e.g., [15,16,17,18,19]). There are studies which show that the mechanical parameters of identical concrete specimens can differ up to 20% (e.g., [20]) due to a lack of control of the fibre fraction of the concrete. For this reason, large safety factors must be used for static calculations.

While the fibre content in new construction projects can easily be determined by washing fibres out of a defined fresh concrete sample or by checking the weighing protocols, in hardened concrete or existing constructions, to date, there is no easy method to calculate the exact fibre content. One non-destructive test method that seems to be suitable for the determination of fibre content and orientation in concrete is to measure the electrical resistance of the concrete (e.g., [21,22,23,24,25,26,27,28,29,30,31]). Resistance measurements with so called multiring-electrodes are well known to monitor the durability of concrete, with respect to potential corrosion processes of steel rebars (e.g., [32,33,34,35]).

The degree of water saturation of the pore structure of the concrete, the porosity and the conductivity of the pore solution are the most relevant influencing factors for the electrical resistivity of concrete (e.g., [26,36,37,38]). Nevertheless, it should be considered, that the influence of the pore solution chemistry is relatively small, unless extreme drying and carbonating effects take place. In general, the composition of the pore solution changes with the ongoing hydration process due to hydration reactions and related dissolution processes of ions, especially during early age until 28 days of hydration. Typically, the chemical composition stabilises afterwards and shows an electrical resistivity in a range between 0.3 and 1 Ωm (e.g., [37,38]).

For SFRC, the fibres have a significant influence on the conductivity of the material due to their ability to function as metallic conductor in contrast to the pore solution which only conducts electricity via ionic transfer (e.g., [11,24,34,39]). Therefore, for the concrete, if investigated under controlled humidity, especially the pore structure influences the resistivity and in turn is influenced by the process of hydration and the concrete composition (e.g., [40]). By screening the literature several other parameters become apparent, which affect the pore structure of concrete, such as water/cement ratio, cement content and the concrete ingredients, e.g., type of cement or applied supplementary cementitious materials and aggregates (e.g., [41,42,43,44,45,46,47,48,49,50,51]).

The most influencing parameters on the porosity and, therefore, on the electrical resistivity of concrete are the water/cement ratio and the cement content. An increasing water/cement ratio is paired with an increase in porosity; therefore, a decrease in electrical resistivity due to an enlarged capillary network is observed. The total amount of water, at a constant w/c ratio, is influenced by the cement content, which also influences the total porosity. At constant cement content, an almost linear decrease in electrical resistivity is visible with increasing water/cement ratios between 0.4 and 0.6, while higher cement contents at a constant w/c ratio also lead to decreasing electrical conductivity. Both parameters are independent of each other and showed no interactions (e.g., [41,44,45,46,47,48,49,50,51]).

Different types of cement as well as the use of SCM significantly affect the conductivity of the pore solution as well as the resulting pore structure of the concrete. In general, ordinary Portland cements show the lowest electrical resistivity, while the use of different cement types or the addition of silica fume, fly ash or ground granulated blast-furnace slag (GGBS) significantly decreases the porosity of the concrete, thus leading to an increase in electrical resistivity. Mainly due to the pozzolanic or latent-hydraulic reaction of SCMs, such as GGBS, silica fume or fly ash the pore structure becomes finer and less permeable, and thus the conductivity of the pore solution is reduced. The same phenomenon can be observed using finer cements, such as cements with higher strength classes (e.g., [42,46,52,53,54,55,56,57,58,59,60]).

Additionally, aggregates may influence the conductivity of concrete, since different grain size distributions or water absorption capacities of these concrete components can influence the total porosity of the concrete. For example, the use of crushed aggregates leads to a higher electrical resistivity than round grain shapes. Additionally, a greater maximum grain size increases the electrical resistivity as large grains are non-conducting and increase the tortuosity of the concrete (e.g., [41,61]). It must be mentioned that in some cases conductive aggregates are also used for concrete, which leads to a significant decrease in the electrical resistivity.

One additional parameter that may have a large effect on the electrical resistivity independent of the concrete composition is the aging of the concrete as a function of service exposure, which is paired with changes in the pore structure and also impacts leaching and drying processes. Furthermore, the continuing hydration process leads to a refinement and densification of the pore structure, thus increasing electrical resistivity. The same effect occurs due to ion leaching effects (e.g., [55,62,63,64]).

When studying SFRC, the use of conductible fibres, e.g., steel, also contributes to electric conduction processes and thus significantly affects the electrical resistivity of the composite material. Since steel is a metallic conductor, the fibres have a substantially higher conductivity than the concrete. Therefore, by the addition of steel fibres, a composite is formed, which includes a combination of parallel and serial electrical connections. Thus, the electrical resistivity of the composite is dependent on the amount of fibre and orientation in the structure. A high fibre content leads to a significant decrease in electrical resistivity, and fibres with a parallel orientation to the electrical transport direction reduce resistivity. In addition, the geometry of the fibres influences conductivity. Small fibres, in general, tend to show an even distribution and orientation, whereas the use of longer fibres increases the probability of direct fibre to fibre contacts, which leads to a short circuit and, therefore, to almost no influence of the concrete on the electrical conductivity. These effects of fibre length, however, only take place at low fibre dosages (e.g., [24,39,65]).

Measurements of the electrical resistivity of the concrete show a high potential for use as non-destructive test method for the determination of the fibre content of SFRC, and thus possible negative effects of other parameters have been known to enable sufficient and accurate results by the use of an easy test setup. Even though, in the past, investigations have been performed to show the influence of fibres on the electrical resistivity (e.g., [21,22,23,24,25,26,27,28,29,30,31]) or to analyse effects of the concrete composition on the permeability or electrical resistivity of concrete (e.g., [37,38,39,40,44,45,47,48,49,50,51,65]), a systematic study that combines the effects of SFRC and different parameters of the concrete composition is lacking.

In this study, the effects of concrete composition in combination with the addition of fibres on the electrical conductivity are studied. The aim of this paper is to identify single effects and interactions which allow a model to be constructed and/or represent an important dataset for the future development of a test method for the determination of fibre content and orientation on fresh and hardened concrete based on electrical resistivity measurements. With the help of such a novel test method, it is possible to simplify monitoring processes for SFRC and to allow a more precise prediction of the mechanical characteristics of SFRC, which could lead to static calculations with lower safety factors and thus to a higher acceptance of SFRC in fields of construction.

## 2. Materials and Methods

### 2.1. Materials

#### 2.1.1. Characterisation of Materials

For the experiments presented in this paper, we decided to use three different ordinary Portland cements originating from the same cement plant with strength classes of 32.5 N/mm^2^, 42.5 N/mm^2^ and 52.5 N/mm^2^, as these types are commonly used, and neither negative interactions based on chemical reactions in combination with ground granulated blast-furnace slag (GGBS) nor changes in clinker composition for the different cements should take place. Additionally, the use of GGBS was investigated in different mixtures. Quartzite gravel with a grain size distribution of A/B 16 in accordance with DIN 1045-2 [66] was the used aggregate.

The binder components, cements and GGBS, were analysed regarding density and fineness to consider these factors in the evaluation of the measurements of the electrical resistivity. The results are presented in Table 1.

All three cements showed almost the same density, while the GGBS had an 8% lower density. This difference was taken into account for the concrete mix design. The cements were selected to differ in fineness, so the results of specific surface according to Blaine optimally fulfilled this requirement. The specific surface of the CEM I 52.5 R exceeded that of the CEM I 32.5 R by 44%, and the specific surface of CEM I 42.5 R, which is 22% higher than that of the CEM I 32.5 R, almost presented the mean value of the two other cements. The specific surface of the GGBS, as expected, exceeded that of the less fine cements and was in the same range as the CEM I 52.5 R.

**Figure 1:**
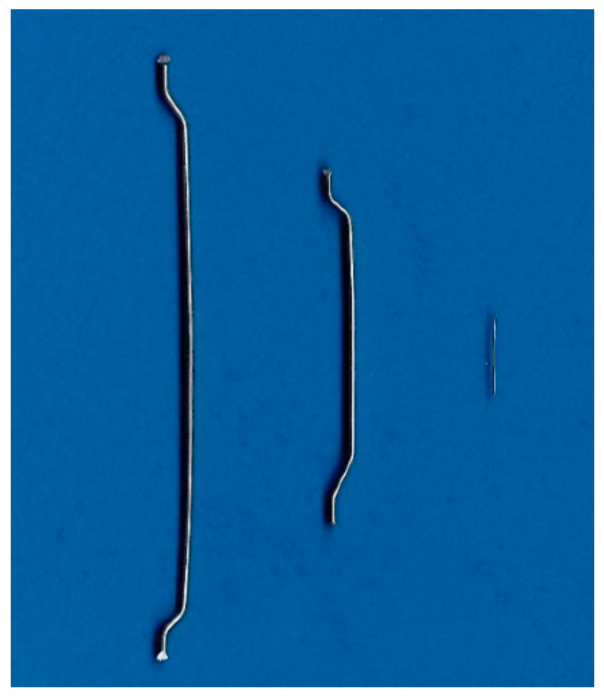
Shapes of the used fibres (left: fibre 1; middle: fibre 2; right: fibre 3).

Three different steel fibres were used as the reinforcement materials for the experiments. While fibre 1 and fibre 2 are hooked-end macrosteel fibres, produced as wired fibres, fibre 3 is a straight microsteel fibre with a brass coating. Fibre 1 represents a type of steel fibre that is commonly used in Europe for SFRC. In contrast, fibre 3 is recommended for use in UHPC, as presented in [67]. Fibre 2 was chosen due to its geometry, which is directly in between those of the other fibres. The fibres differ in length, diameter and aspect ratio, but the greatest difference is the fibre quantity. While there are 2600 fibres of type 1 per kg, the quantity of fibre 3 with 662,000 fibres is almost 250 times higher. A characterisation of the fibres is given in Table 2, and a graphical presentation of the fibres is presented in Figure 1.

#### 2.1.2. Basic Concrete Mix Design

The electrical conductivity of both plain concrete (PC) and steel fibre-reinforced concrete (SFRC) was investigated on various concrete mixtures to identify the effects of different parameters of the concrete composition on electrical resistivity. For this purpose, a basic concrete mixture according to EN 206 [68] in combination with DIN 1045-2 [66] was chosen and investigated.

The basic concrete composition is given in Table 3. A cement content of 300 kg/m^3^ of CEM I 32.5 R in combination with a water/cement ratio of 0.60 was used to obtain a sufficient workability with the use of a high amount of steel fibres. For the reinforcement, fibre 1 (Table 2) was chosen, and fibre contents from 0 kg/m^3^ to 80 kg/m^3^ were applied. No superplasticizer was used.

Every concrete batch within this study was produced according to the following scheme with a material volume of 60 L. The homogenisation of the solid materials, such as cement, GGBS and aggregates, was performed in a compulsory mixer with a nominal volume of 160 L for 30 s. In the next step, the water was added during the ongoing mix process, and the concrete was mixed for at least two minutes. After a visual inspection of the homogeneity of the concrete, adhering components were removed from the mixer wall, followed by an additional mixing time of one minute.

Directly after the mixing process, the consistency of the concrete was determined via a flow table test in accordance with EN 12350-5 [69]. Additionally, the air content and fresh concrete density in accordance with EN 12350-6 [70] and EN 12350-7 [71], respectively, were determined. After the execution of the fresh concrete tests, samples of 4 L of concrete were placed in a small bucket mixer with a mixing volume of a maximum of 10 L. Defined amounts (between 10 kg/m^3^ to 80 kg/m^3^) of steel fibres were added into the bucket mixer, and the fresh concrete was mixed for approximately one minute to ensure the correct fibre amounts. Then, cubical steel formworks with dimensions of 150 mm^3^ were filled with the SFRC. One additional reference specimen was produced using the plain concrete without fibres.

After the production, the specimens were left in the formworks for approximately 24 h and covered with foil to prevent drying of the surfaces. The upper surfaces of the demoulded specimen were removed by grinding since a flat surface was recommended to reach optimal conditions for the electric connection. Afterwards, the dimensions of the specimens were determined, and they were stored in 2.7 L of regular water each at a temperature of 20 °C in separate storage boxes till testing.

In total, 180 specimens were investigated, including the basic concrete mixture, ten additional mixtures for the single parameter variation and eight mixtures for the multiple parameter variation with nine specimens each and nine specimens for the investigations of possible leaching effects. 

### 2.2. Experimental Methods

#### 2.2.1. Design of Experiments—Single Parameter Variation

For the identification of changes in single parameters of the concrete mix design, several mixtures with one varying parameter were produced and investigated. The following parameters were varied individually in order to prevent interactions based on combined variations:

- Cement type (CEM I 32.5 R, CEM I 42.5 R and CEM I 52.5 R) (all from the same plant);
- Water/cement ratio (0.55, 0.60 and 0.65);
- Binder content (270 kg/m^3^, 300 kg/m^3^ and 330 kg/m^3^);
- Content of GGBS (replacement of 0%, 35% and 65% of total binder);
- Fibre type/geometry (macrosteel fibre 60 mm, macrosteel fibre 35 mm and microsteel fibre 8 mm).

In total, based on the basic concrete mix design, ten additional concrete mixtures were produced and investigated with fibre contents from 0 kg/m^3^ to 80 kg/m^3^, so each of the 5 parameters could be observed at three levels to identify possible effects and evaluate if there is a linear or non-linear correlation. The mix designs of the additional concretes are presented in Table A1, Table A2, Table A3, Table A4 and Table A5, Appendix A.

#### 2.2.2. Design of Experiments—Multiple Parameter Variation

To identify interactions between different parameters of the concrete composition, the software Minitab (19.2020.1, Minitab LLC, State College, PA, USA) was used to develop a statistical design of experiments. Based on the basic concrete mix design (Table 3), the cement type of the parameters, the w/c ratio, the binder content and the amount of GGBS were varied, and a matrix of 9 mixtures was investigated (Table 4). The fibre type was kept constant in these experiments to keep the number of mixtures as small as possible, and no large effects of the fibre type on the electrical resistivity were identified in the experiments with single parameter changes. An evenly distributed matrix of mixtures was important, so that each parameter at each of its three tested levels is presented three times within the complete evaluation. The mix designs of the additional concretes are presented in Table A6, Table A7, Table A8 and Table A9, Appendix A.

#### 2.2.3. Investigations of Leaching Effects

In addition, the possible impact of leaching of the concrete during its storage was investigated. To identify this effect, one series consisting of nine specimens out of one batch of concrete was produced as described in Section 2.1.2. Samples produced from the basic concrete mix with fibre contents of 0 kg/m^3^, 40 kg/m^3^ and 80 kg/m^3^, three almost identical specimens of each fibre content, were tested. Directly after demoulding, the specimens were stored under varying conditions. One specimen of each fibre content was stored in water as described above, while the other two specimens of each fibre content were exposed to alkaline solutions with higher pH to minimise leaching effects over time. Among these solutions was saturated calcium hydroxide solution, and another was synthetical pore solution with the composition presented in Table 5. The specimens were only taken out of their storage boxes for the measurements at concrete ages of 7 days, 14 days, 28 days, 56 days, 91 days and 185 days and afterwards stored in the same boxes again without a change in the storage medium with the aim of the harmonisation of the concrete and storage medium over time. The volume to surface ratio of all storage media was constantly 20 L/m^3^.

### 2.3. Resistivity Measurements

**Figure 2:**
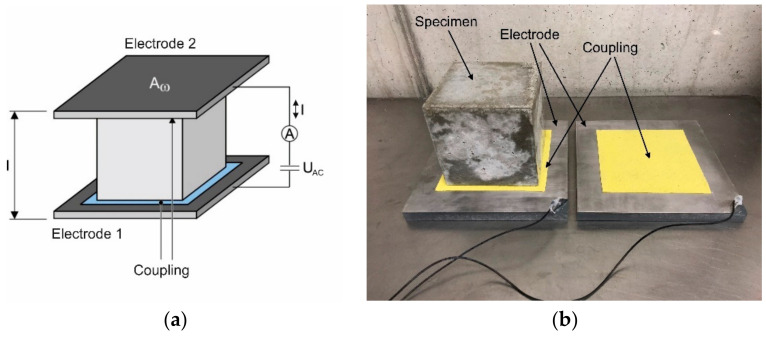
Test setup for conductivity measurements, schematical setup (**a**) and photo of test setup with investigated specimen (**b**).

Each specimen was taken out of the storage boxes at a concrete age of 7 days, and the electrical resistance between two opposite surfaces of the specimen was measured under wet surface conditions using an alternating current. The aim was to contact the whole surface due to the large ratio between the fibre length and dimensions of the specimen to inhibit the effects of single fibres or grains near the surfaces. To consider effects based on the inhomogeneity and anisotropy of the concrete, especially in the case of fibre addition, all three directions, two horizontal ones and the vertical direction, which is the pouring direction, were contacted consecutively using an LCR meter. Additionally, every direction was investigated with five different frequencies of alternating current, 100 Hz, 120 Hz, 1 kHz, 10 kHz and 100 kHz, with a voltage amplitude of 600 mV rms. The test setup is illustrated in Figure 2.

The whole test setup consisted of two stainless steel plates with edge lengths of 200 mm. Due to the uneven and porous surface of the concrete, a direct electrical connection of steel and concrete led to irreproducible results; therefore, a wet sponge cloth was used as a connector between both materials, and the humidity of the sponge was constantly checked to inhibit drying effects. The steel plates were directly connected to the LCR meter via equally long cables. After placing the specimen between both electrodes, a defined reference weight was placed on top of the test setup to gain a constant and reproducible contact pressure.

Since the dimensions of the specimens slightly differed, the resistivity of the concrete for each direction was calculated by Equation (1):

ρ = R × k(1)

with 

ρ: electrical resistivity in Ωm

R: electrical resistance in Ω

k: geometry factor depending on dimensions of specimen in m (Equation (2))

k = A/l(2)

with 

A: effective contact area in m^2^

l: electrode gap in m

Due to the inhomogeneity and anisotropy of SFRC, it was necessary to carry out several measurements in three possible specimen directions. For this purpose, the resistivities of the three directions for each specimen were averaged, and the so called global electrical resistivity ρ,g was used to compare different specimens. Values for different directions were examined in detail in only a few instances. Even though the whole study was performed using a constant production method, effects based on the compaction, manufacturing of specimens and flowing behaviour of concrete and fibres would result in a considerable scatter of material characteristics, especially in fibre orientation and distribution, so the use of a global value independent of specimen orientation would ideally minimise this scatter. The calculated global electrical resistivity of the material represents the approximated electrical resistivity of an equivalent material with homogenous and isotropic behaviour in electrical conduction. All results of the calculations of the global electrical resistivity can be found in Table A10, Table A11, Table A12, Table A13, Table A14, Table A15, Table A16, Table A17, Table A18, Table A19, Table A20, Table A21, Table A22, Table A23, Table A24, Table A25, Table A26, Table A27, Table A28, Table A29, Table A30, Table A31, Table A32, Table A33, Table A34, Table A35, Table A36, Table A37, Table A38, Table A39, Table A40, Table A41, Table A42, Table A43, Table A44, Table A45, Table A46, Table A47, Table A48, Table A49, Table A50, Table A51 and Table A52, Appendix A.

## 3. Results

### 3.1. General Results of the Evaluation

**Figure 3:**
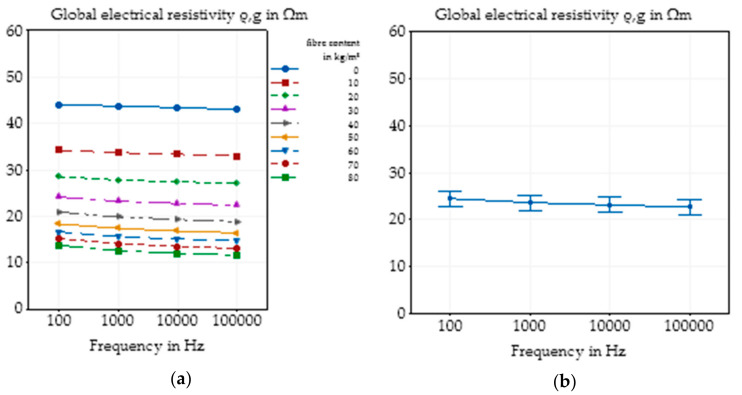
Effect of different frequencies of alternating current on the global electrical resistivity of SFRC as function of the fibre content in the basic concrete mixture (**a**) and main effect plot with 95% confidence interval of different frequencies of alternating current on the global electrical resistivity of SFRC calculated by Minitab based on all experimental data (**b**).

In the first step, the influences of the frequency of the alternating current as well as the fibre content on the global electrical resistivity were analysed based on the results of basic concrete mixture. As seen in Figure 3a, an increasing frequency, as expected, led to a small decrease in the global electrical resistivity independent of the fibre content. Based on all 180 specimens, independent of concrete composition and fibre content, the main effect plot also shows the decrease in the electrical resistivity with a higher frequency, where a linear tendency is visible for a logarithmic increase in the frequency (Figure 3b). In addition, it can be seen that there was an almost constant 95% confidence interval for all frequencies. The main effect plot was calculated by Minitab by compiling the mean values of different groups of results to identify effects that are only caused by one parameter. In comparison to the influences of the concrete composition and the fibre addition, this tendency was scarce, so for this study, it could be neglected. Due to the independency of the alternating current and all other investigated parameters, which can be presumed based on the main plot by Minitab, for further investigation, all the measurement results of the five frequencies were included in the evaluation, and the software Minitab was used to calculate the main effect plots to eliminate the effect of frequency and enlarge the database.

**Figure 4:**
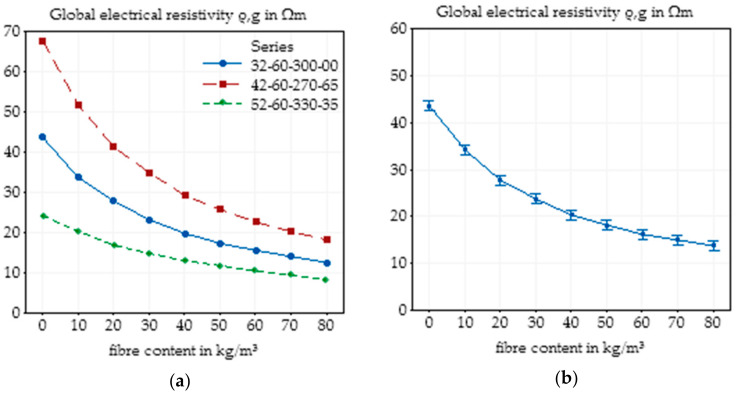
Effect of the fibre content on the global electrical resistivity of SFRC in the basic concrete mixture (**a**) and main effect plot with 95% confidence interval of the fibre content on the global electrical resistivity of SFRC calculated by Minitab based on all experimental data (**b**).

Additionally, as shown in Figure 4a, where the basic concrete mixture and the mixtures with the maximum and minimum electrical resistivity, respectively, are presented, the content of fibres in SFRC had a huge effect on the electrical resistivity. A higher fibre content, for this mixture, led to a lower resistivity. The same behaviour was visible for the main effect plot, including all specimens with different concrete mixtures and the 95% confidence interval (Figure 4b), which almost exactly presented the basic concrete mixture, indicating that the parameter variations led to both increasing and decreasing global electrical resistivities. With increasing fibre content, the difference in the electrical resistivity of varying concrete mixtures reduced in size. While the absolute difference of the maximum and minimum electrical resistivity for the plain concrete was 43.2 Ωm, that for a fibre content of 80 kg/m^3^ with 10.7 Ωm was significantly lower; however, the confidence interval for the mean values remained almost constant. Relative to the mean values, the maximum and minimum spread was 99% for the plain concrete and 71% for the SFRC with 80 kg/m^3^ of fibres.

In contrast to the frequency of alternating current, the influence of different fibre dosages was significantly higher, and a non-linear correlation between fibre content and global electrical resistivity was evident. As interactions between the fibre content and other parameters of the concrete composition were not visible and also not expected to take place, Minitab was used to calculate the main effect plots of the varied parameters to easily compare changes in concrete composition without the effects of fibres. Until an interaction between concrete composition and fibre content cannot be ruled out, especially due to the charge transport in the steel concrete interface, in the following chapters of this paper, the influence of each parameter is examined individually for each fibre content.

### 3.2. Effects of Single Parameter Variations in Concrete Composition

#### 3.2.1. Changes in Water/Cement Ratio

**Figure 5:**
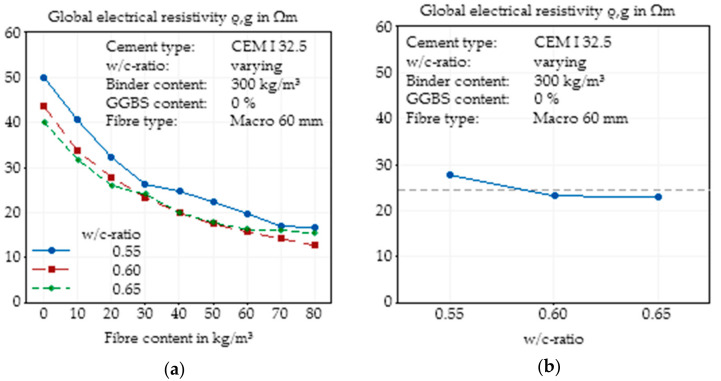
Effect of the water/cement ratio on the global electrical resistivity of SFRC as a function of fibre content (**a**) and main effect plot of the water/cement ratio on the global electrical resistivity of SFRC calculated by Minitab (**b**).

The basic concrete mix design (Table 3) was modified with respect to its water/cement ratio. As shown in Figure 5a, for all fibre contents, there was a visible influence of the w/c ratio on the resistivity, and an increasing fibre content resulted in a significant reduction in the global electrical resistivity. While a w/c ratio of 0.55 led to the highest global electrical resistivity for all fibre contents, the higher w/c ratios of 0.60 and 0.65 did not show an equally continuous tendency. The curves for w/c ratios of 0.55 and 0.60 can be characterised through an almost parallel shift, but the curve for a w/c ratio of 0.65 showed a different behaviour and a smoother progression. It is apparent that at w/c ratios greater than 0.6 and at a fibre content higher than 30 kg/m^3^, a plateau was reached, at which an increase in the w/c ratio did not significantly impact the resistivity.

In summary, independent of fibre content, an increase in the w/c ratio leads to a decrease in resistivity or to an increase in conductivity, due to the higher water amount in concrete and the enlarged porosity. This effect already is described for plain concrete in several papers (e.g., [44,45]). Nevertheless, there is no linear correlation between the w/c ratio and resistivity, especially when fibres are added. For the higher fibre contents, the discrepancy between the expectation and results of the measurement can be explained by the influence of fibres and the lower resistivities. While for low fibre contents, the concrete had a huge influence on the electrical resistivity, with increasing fibre content, the fibres progressively became the main conductive part of the material. Variations of the fibre distribution and orientation based on slightly varying flowabilities of the concrete can have a significant influence on the global electrical resistivity of the SFRC.

#### 3.2.2. Changes in Binder Content

**Figure 6:**
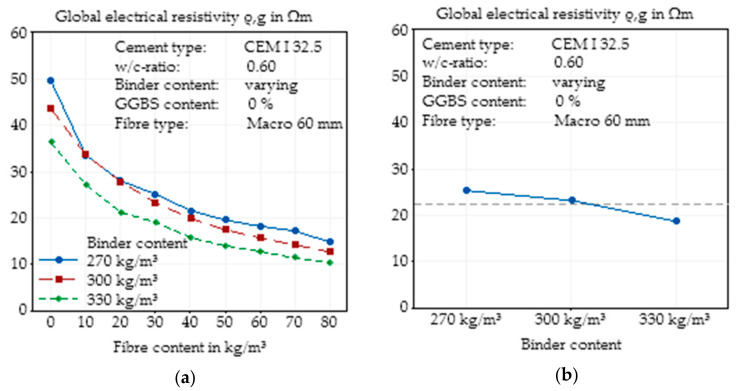
Effect of the binder content on the global electrical resistivity of SFRC as a function of fibre content (**a**) and main effect plot of the binder content on the global electrical resistivity of SFRC calculated by Minitab (**b**).

In addition to the water/cement ratio, the binder content, which in this case was synonymous to the cement content due to the lack of SCM in the basic concrete mix design, directly influenced the total amount of water and hydrated cement paste in the concrete mixture. Hence, the total porosity of the concrete varied, whereas the pore size distribution was only marginally impacted. Figure 6a clearly shows the expected effect (e.g., [45,47]) of a higher amount of water, which led to a decrease in electrical resistivity for almost every fibre content. The curves for all cement contents in Figure 6a show almost the same behaviour with a decrease in electrical resistivity with increasing fibre content and a flattening of the curve for higher fibre contents. Only the concretes with a cement content of 270 kg/m^3^ and fibre contents of 10 to 30 kg/m^3^ seemed to deviate from the constant decreasing behaviour, but this can be explained by the scatter of the experiments.

In Figure 6b, the total effect of the variation of the cement content independent of fibre content is presented. For the investigated cement contents, an almost linear correlation between cement content and electrical resistivity can be observed. The constant w/c ratio in this series of experiments caused a change in the total amount of cement stone with presumably no changes in the pore structure. Based on the isolating properties of the aggregates, this is paired with an increase in the conductivity of the whole material.

#### 3.2.3. Changes in Cement Type

**Figure 7:**
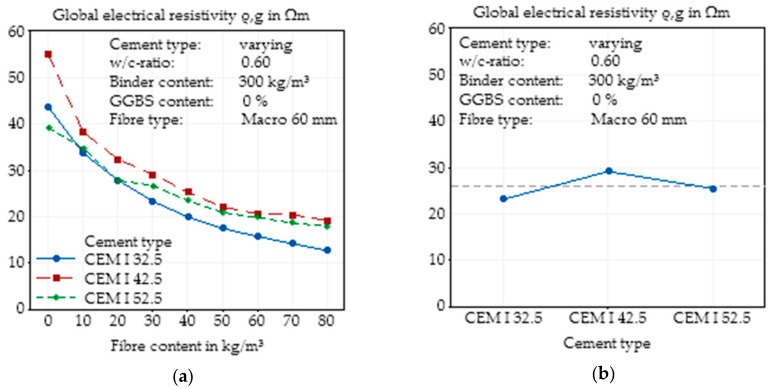
Effect of the cement type on the global electrical resistivity of SFRC as a function of fibre content (**a**) and main effect plot of the cement type on the global electrical resistivity of SFRC calculated by Minitab (**b**).

The cement type, in this case, the strength class, synonymous with the specific surface of the cement, was varied to identify the effects of a finer pore structure of cement on higher strength classes. Comparing the results of CEM I 32.5 R and CEM I 42.5 R, this expected behaviour could be observed, but the finest cement (CEM I 52.5 R) does not fit into those expectations (Figure 7a). As described in Section 2.1.2., the specific surfaces of the three used cements were determined, showing near linearity, where CEM I 32.5 R showed the lowest surface and CEM I 52.5 R the highest surface, and the specific surface of CEM I 42.5 R was approximately the mean value of both. When the curves of the global electrical resistivities of the three concrete mixtures, it is apparent that especially for small fibre contents, the cement with the highest surface showed the lowest electrical resistivity. For higher fibre contents, contrary expectations, the curves of the cement intersected and the resistivity of CEM I 52.5 R exceeded that of CEM I 32.5 R, but they were still lower than those of CEM I 42.5 R. These effects could not be explained and will be addressed in a future analysis.

In Figure 7b, the effect of the cement type on the electrical resistivity is presented independent of the fibre content. As already described, the expected tendency of a higher resistivity with a higher fineness of cement is only visible for the two lower strength cements, and the results of the CEM I 52.5 R are much lower than expected. A plausible explanation could be the slightly different compositions within the cement clinker, although all cements were produced in the same production plant.

#### 3.2.4. Addition of Ground Granulated Blast-Furnace Slag (GGBS)

In addition to the different cement strength classes, GGBS was used as a concrete component due to its well-known ability to significantly alter the pore structure of the concrete. To identify the effects of GGBS without any interactions with different cement clinkers, the same base cement (CEM I 32.5 R) was blended with GGBS at a constant water/binder ratio. The contents of GGBS were chosen considering the limits for additives for slag cements.

**Figure 8:**
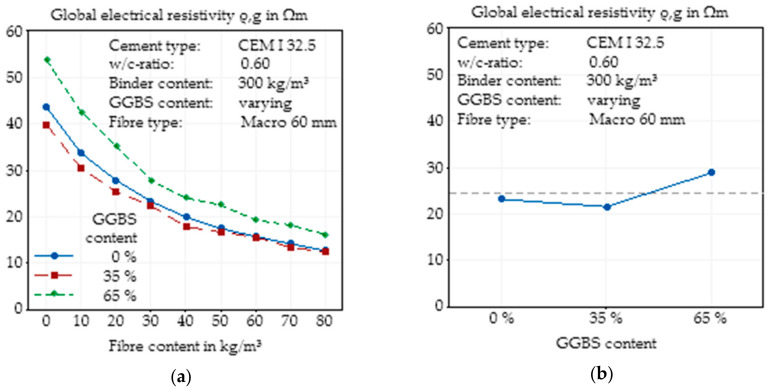
Effect of the addition of GGBS on the global electrical resistivity of SFRC as a function of fibre content (**a**) and main effect plot of the addition of GGBS on the global electrical resistivity of SFRC calculated by Minitab (**b**).

The use of a GGBS content of 35% of the total binder content did not result in any significant changes in the electrical resistivity (Figure 8a). For small fibre dosages, the OPC concrete had a slightly higher resistivity, while the difference decreased with increasing fibre content. In contrast, using a GGBS content of 65% led to a significant increase in the electrical resistivity, where the difference again decreased for higher fibre contents.

The summarising main effect plot (Figure 8b) shows that a GGBS content of 35% led to almost no changes in the conductivity of the concrete independent of the fibre content. It can be concluded that after 28 days of hydration, there were only small effects on the pore structure due to the addition of 35% of GGBS, and the conductivity of the pore solution was only marginally influenced by the resulting changes in the chemical composition of the pore solution. The addition of a high content of GGBS showed almost the same effect as using a finer cement. The resulting finer pore structure, which was expected by the addition of GGBS (e.g., [49,50]) and thus a reduced transmissibility of the pore solution, which functions as an electrical conductor, was caused by a higher resistivity than the reference sample. Earlier investigations (e.g., [45,51]) have already shown that, for plain concrete, the addition of GGBS leads to an increase in electrical resistivity.

#### 3.2.5. Effects of Multiple Parameter Variations

**Figure 9:**
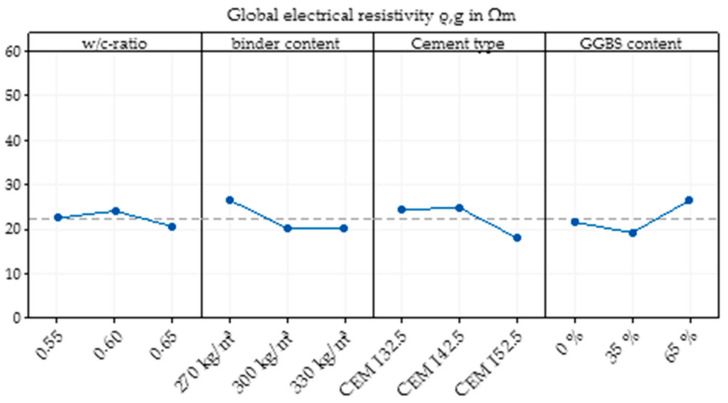
Main effect plot of the concrete composition on the global electrical resistivity of SFRC calculated by Minitab based on the mixtures of the multiple parameter variation.

A statistical design of experiments was used to detect possible interactions among different parameters to check the possibility of the superposition of the single effects of different parameters on the global electrical resistivity. Comparing the results of the single parameter variation to the results of the multiple parameter variation, it is clear that there were differences in the main effects of the analysis of Minitab (Figure 9). All four parameters do not show any constant behaviour—neither a continuous increase nor a continuous decrease in the electrical resistivity. 

The results of the first three parameters, w/c ratio, binder content and cement type, of the multiple parameter variation differed from that of the single parameter variations, which shows that a superposition of these parameters is not possible in every case. Only the results of the addition of GGBS content are slightly similar to those of the single parameter variation.

**Figure A1:**
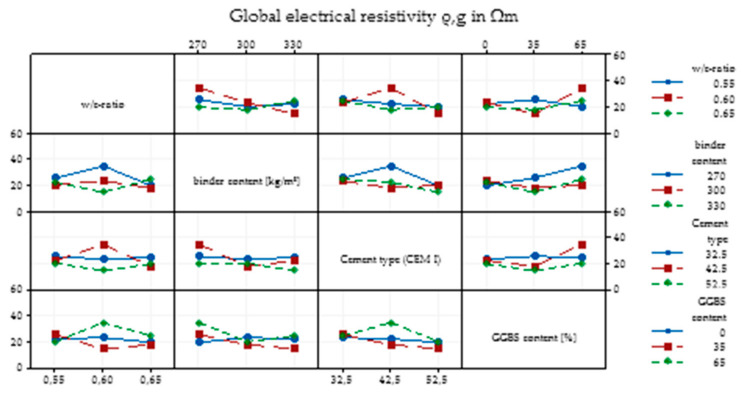
Interaction plot of the effects of the concrete composition on the global electrical resistivity of SFRC calculated by Minitab based on the mixtures of the multiple parameter variation.

In sum, it is clear that the results of single parameter variation and multiple variation completely differ. This result can be confirmed by observing the interaction plot of the multiple parameter changes (Figure A1). There, all possible interactions between changes in multiple parameters with an interaction level of 2 are presented, which means that interactions among the three parameters were not observed. An independency of two parameters would be presented through three curves in one sector that are only shifted in parallel. As can be seen, there is no such sector in the whole plot. Often, only one point did not reflect this behaviour, as with an interaction of cement type and GGBS content, but sometimes, totally different behaviours of the curves were noticeable, as with a combined observation of the w/c ratio and GGBS content (lowest left sector), where, for a GGBS content of 0%, there was almost no influence of the w/c ratio on the resistivity; for a GGBS content of 35%, a decrease, followed by a slight increase, with an increase in the w/c ratio was visible; and for a GGBS content of 65%, the opposite behaviour was observed.

One problem of the statistical design of experiments was the low number of tested concretes, since a full matrix of 81 possible mixtures was investigated and approximated by the analysis of only 9 mixtures. Through the partially low influences of single parameters and the presented interactions, a more detailed analysis of interaction effects was not possible.

### 3.3. Effects of Variation of Steel Fibre Type

#### 3.3.1. Effects of Fibre Type on Global Electrical Resistivity

**Figure 10:**
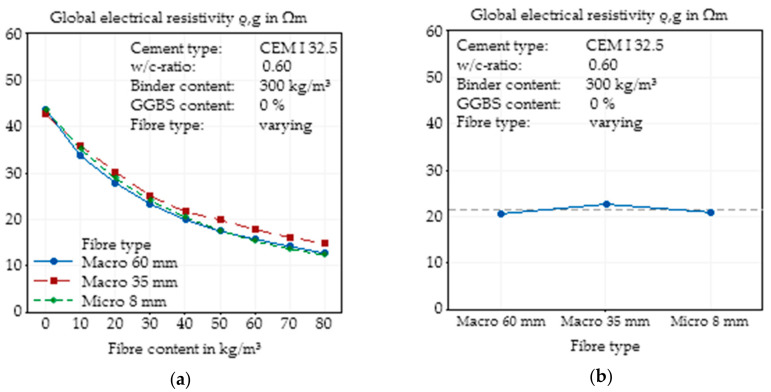
Effect of the steel fibre type (geometry) on the global electrical resistivity of SFRC as a function of fibre content (**a**) and main effect plot of the steel fibre type (geometry) on the global electrical resistivity of SFRC calculated by Minitab (**b**).

The last parameter that was analysed via single parameter variation was the fibre type. Two macroscopic fibres with fibre lengths of 60 mm and 35 mm, respectively, and one microfibre with a fibre length of 8 mm were used. The results of the variation show that there was almost no influence on the electrical resistivity for all three types of fibres. While all three plain concrete mixtures are almost identical, which indicates comparable basic mixtures, with an increasing fibre content, only the addition of the smaller macrofibre (35 mm length) led to a slightly higher electrical resistivity (Figure 10a). The longest and shortest fibre showed almost identical results. Hence, the main effect plot (Figure 10b) shows that all three types of fibres behaved similarly for this concrete mixture. 

#### 3.3.2. Effects of Fibre Type on Electrical Resistivity in Horizontal Direction

**Figure 11:**
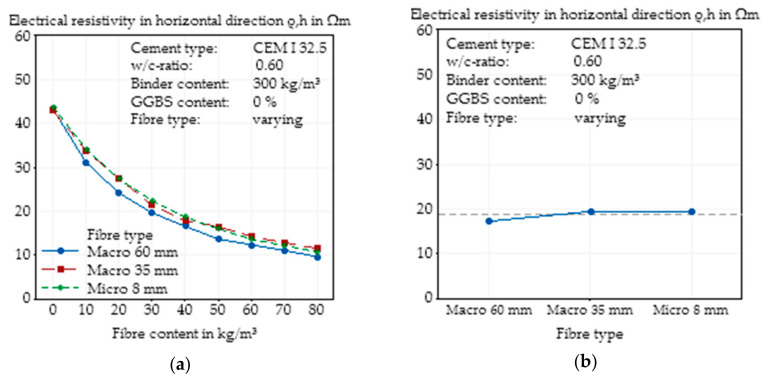
Effect of the steel fibre type (geometry) on the electrical resistivity in horizontal direction of SFRC as a function of fibre content (**a**) and main effect plot of the steel fibre type (geometry) on the electrical resistivity in horizontal direction of SFRC calculated by Minitab (**b**).

While there was almost no visible effect on the global electrical resistivity of the concretes with different types of fibres, possible variations in the distribution of different specimen directions, depending on the fibre type and manufacturing process, were identified, which was performed by the use of a vibrating table. Figure 11 shows the effects of different fibres on the electrical resistivity measured in the two horizontal directions of the specimens. The horizontal directions are those which are orthogonal to the direction of the vibrating table. Analogous to the last chapter, almost no influence was visible for all fibre contents. The longest fibre again led to the smallest resistivities, but there were no significant effects measurable.

Comparing the global electrical resistivity (Figure 10a) and that in the horizontal direction (Figure 11a), it is noteworthy that the electrical resistivity of the SFRC in the horizontal direction is slightly smaller than the global resistivity, and so the slope of the curves in Figure 10a is steeper.

#### 3.3.3. Effects of Fibre Type on Electrical Resistivity in Vertical Direction

**Figure 12:**
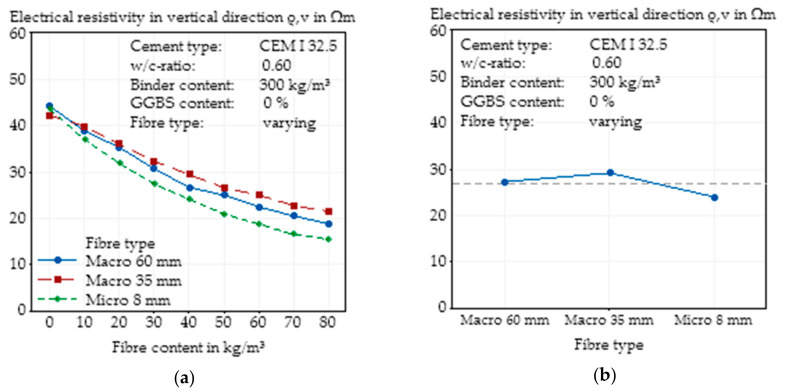
Effect of the steel fibre type (geometry) on the electrical resistivity in vertical direction of SFRC as a function of fibre content (**a**) and main effect plot of the steel fibre type (geometry) on the electrical resistivity in vertical direction of SFRC calculated by Minitab (**b**).

In contrast to the electrical resistivity in the horizontal, the vertical electrical resistivity was significantly higher than the global electrical resistivity, and the spread of the curves of the different fibres was wider than seen for global electrical resistivity or in the horizontal direction (Figure 12a). This phenomenon was expected based on the manufacturing process, where the concrete was filled into the formworks in two layers, and the compaction was performed by a vibrating table in the vertical direction, which led to a more horizontal orientation of the fibres and thus to a higher conductivity in the horizontal direction. For higher fibre contents, the electrical resistivity in the vertical direction of the concrete with microfibres was lower than that of the macrofibres.

Independent of fibre content, a difference between the electrical resistivity of the concretes with the macrofibre with a length of 60 mm and the microfibre in the vertical direction (Figure 12b) was clear, while the global electrical resistivity was almost equal. This fact suggests a preferred orientation of the microfibres in the vertical direction, although the compaction was performed vertically. For the microfibre, the difference of the resistivity between the horizontal and the vertical electrical resistivity was rather small, at lower than 5 Ωm, while there was a significant difference for the macrofibres, at higher than 10 Ωm.

### 3.4. Effects of Concrete Age and Storage Medium

**Figure 13:**
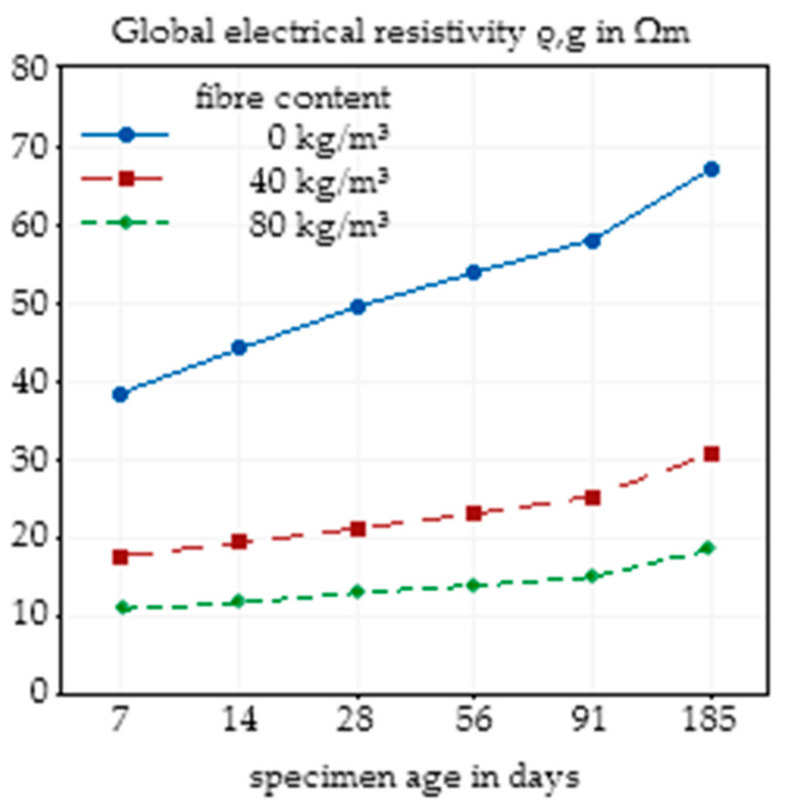
Effect of the concrete age on the global electrical resistivity of SFRC depending on the fibre content based on nine specimens.

In addition to the investigated influencing factors, concrete composition and fibre content, the age of the concrete and the storage conditions can influence the electrical resistivity of the concrete. To show the effects of these factors, specimens from the same mixing batch were used. The influence of concrete age, independent of the storage medium, on the electrical resistivity is presented in Figure 13. As can be seen, for all fibre contents, a higher concrete age resulted in a higher resistivity, which can be explained by the forming of a pore structure and closing of conductive pore channels. It is clear that for plain concretes, the age of the concrete has a greater impact on the resistivity than SFRC, since fibres take part in the conduction process, and thus, the influence of the concrete decreases.

**Figure 14:**
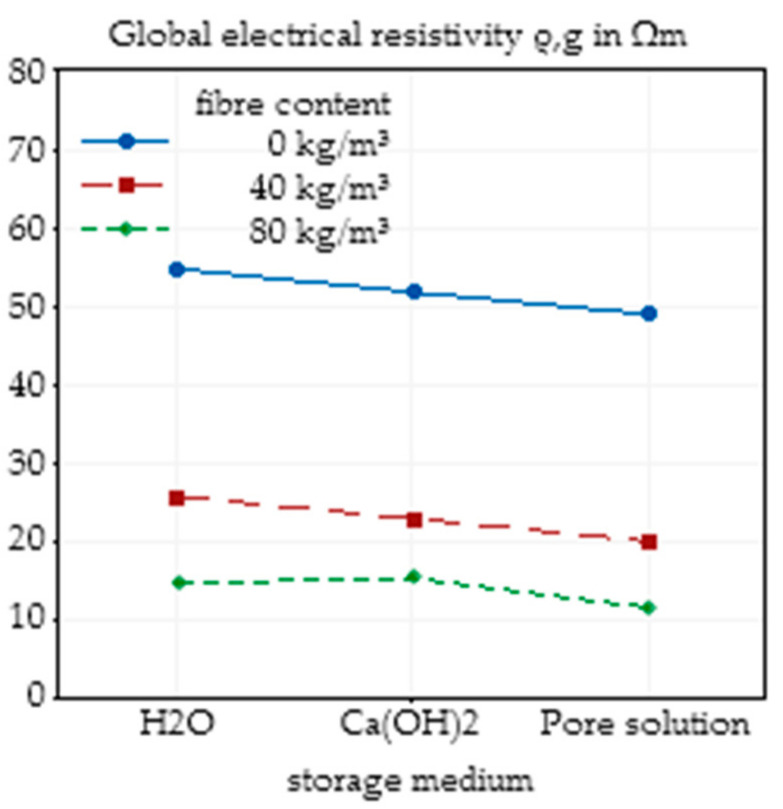
Effect of the storage conditions on the global electrical resistivity of SFRC depending on the fibre content based on nine specimens.

An analysis of the effect of different storage media on the electrical resistivity is presented in Figure 14. From H_2_O over Ca(OH)_2_ to synthetic pore solution, the electrical resistivity decreased, which is in agreement with the increasing initial conductivity of the used storage media. 

**Figure 15:**
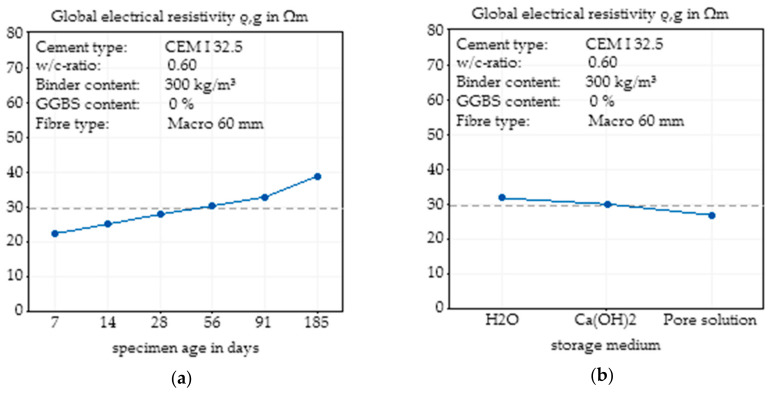
Main effect plot of the concrete age on the global electrical resistivity of SFRC calculated by Minitab based on nine specimens (**a**) and main effect plot of the storage conditions on the global electrical resistivity of SFRC calculated by Minitab based on nine specimens (**b**).

The effect of the age of the concrete and the storage conditions independent of fibre content are presented in Figure 15. As already described, a higher concrete age leads to an increase in electrical resistivity, while storage in a higher alkaline solution reduces resistivity. 

## 4. Discussion

**Figure A2:**
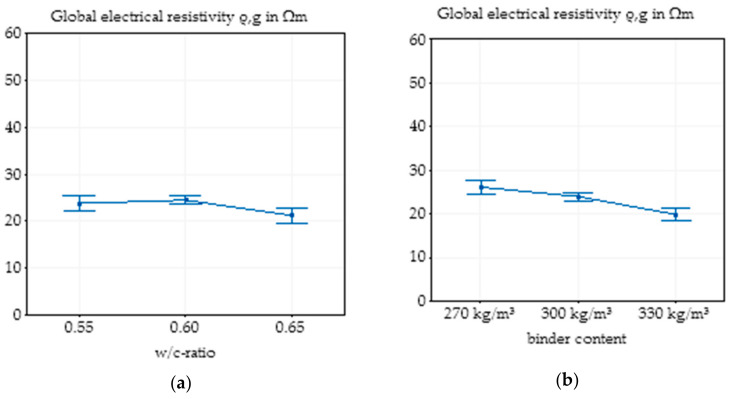
Main effect plot with 95% confidence interval of the w/c ratio on the global electrical resistivity of SFRC calculated by Minitab based on all experimental data (**a**) and main effect plot with 95% confidence interval of the binder content on the global electrical resistivity of SFRC calculated by Minitab based on all experimental data (**b**).

**Figure A3:**
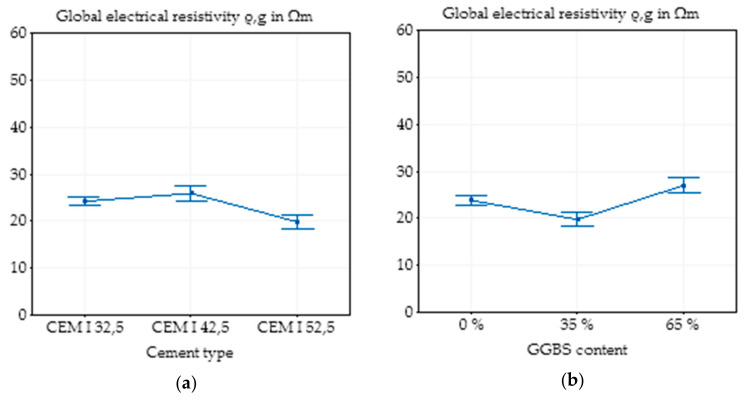
Main effect plot with 95% confidence interval of the cement type on the global electrical resistivity of SFRC calculated by Minitab based on all experimental data (a) and main effect plot with 95% confidence interval of the GGBS content on the global electrical resistivity of SFRC calculated by Minitab based on all experimental data (**b**).

The aim of this study was to identify the relevance of the influencing parameters on the electrical resistivity of SFRC. Hence, variations of the concrete composition as well as changes in storage conditions and concrete age were analysed. To enable the possibility of using electrical resistivity measurements for the detection of the fibre content of SFRC, the fibre content is the most influencing parameter on the electrical resistivity. As presented in Section 3.1, the electrical resistivity, measured with a two-electrode setup, as expected (e.g., [11]), was significantly influenced by changes in the fibre content of steel fibre-reinforced concrete. The average results of all the measurements showed that the addition of 80 kg/m^3^ of fibres resulted in a reduction in the electrical resistivity from approximately 43.5 Ωm to 13.8 Ωm for the mean values, which is equivalent to a reduction of approximately 70% and has the main influence on the electrical resistivity, and no interactions between the fibre content and other varied parameters on the electrical resistivity could be observed. The variation of the concrete composition also led to effects on the electrical resistivity, but compared to the fibre content, the effect is much smaller, which can be seen in Table 6. The percentage influence of all parameters was calculated as the ratio of the minimum to maximum electrical resistivity of the main effect plots (Figure 3b and Figure 4b, Figure A2 and Figure A3). Since a superposition of the single effects of the plain concrete mix design was not applicable (Section 3.2.5), the influence of the fibre content exceeded that of the concrete mixture by at least a factor of 2.5 and of the fibre type by a factor of 6.

As steel fibres are metallic conductors, their conductivity exceeds the ionic conductivity of concrete pore solution, even if the concrete is fully saturated, and so the huge impact of the amount of steel fibres that was identified can be explained. In addition, a high amount of fibres leads to a higher permeability of the concrete (e.g., [5]). For a lower saturation of the concrete, the difference between the conductivity of fibres and concretes may be enlarged significantly, but the measurement of the electrical resistivity will be much more complicated due to coupling effects on the electrodes. Therefore, the large difference in the conductivity allows the use of electrical resistivity measurements to characterise the fibre content of a well-known concrete composition. Since there are additional influencing factors on electrical resistivity, which result from the concrete composition and the age of the concrete, it is necessary to determine the electrical resistivity of the concrete. For this purpose, these parameters were analysed to find possible interactions with the fibres regarding resistivity and to gain a database for the future use of a test setup for the determination of the fibre content of SFRC.

The use of different fibre types, two macrofibres with different lengths of 60 mm and 35 mm and one microfibre with a length of only 8 mm, which was coated with brass, showed almost no effects on the global electrical resistivity of the concretes (Section 3.3.1). This indicates that the fibre content of SFRC can be detected almost independently from the fibre that is used. This fact can be explained by the huge difference between the conductivity of the fibre material and the pore solution of the concrete. Even differences in fibre material only led to nonrecognisable effects on conductivity compared to the high electrical resistivity of the plain concrete. Effects of the fibre length were only expected for small fibre contents in several studies (e.g., [24,39,65]) due to the possible phenomenon of direct connections of two or more fibres. However, as shown in our experiments, even for a fibre content of 10 kg/m^3^, which is uncommon in practice, no such influences could be detected. In summary, it can be concluded that the electrical conductivity of SFRC is mainly influenced by the amount of fibres, respectively, steel and not by the number of fibres, so differentiation between a continuous conductive path via one fibre or a discontinuous conductive path via a plurality of fibres and concrete with transition zones between both materials is not possible.

The only effects of different fibres that were observed resulted from an expected slightly varying distribution and orientation of the microfibres through the mixing and compacting processes. Due to the limited specimen dimensions, long fibres can freely align in the formwork but are extremely influenced by the production procedure. Since the concrete formworks were filled in accordance with European standards, two layers of concrete were filled in and long fibres tended to align more horizontally. In addition, the compaction process via the vibrating table led to a vertical compaction force and thus also supported a horizontal orientation of the fibres. On the contrary, microfibres are sufficiently small to align in the concrete almost freely and are mostly not influenced by aggregates, manufacturing processes in layers or vertical compaction. However, a difference between the macrofibres could not be observed. Therefore, it can be concluded that there is a critical fibre length and a number of fibres per kg, which leads to effects on the fibre orientation, and when this critical value is not reached, no influences will be visible.

The age of concrete, in the investigated range of 7 days to 185 days, led to changes in the electrical resistivity between 40 Ωm and 70 Ωm for plain concrete, and 10 Ωm and 20 Ωm for SFRC, but again, the effects of fibre addition dominated the influence of concrete age. These results confirm the observations of [11], where effects of concrete age, fibre addition and w/c ratio were also investigated. In the first days and months of concrete life, the reaction process caused significant changes in the degree of hydration and thus in the pore structure. Based on the binder material, the greatest part of the hydration was completed after 28 days to 90 days. Hence, the effect of the concrete age on the electrical resistivity decreases with increasing age, which is synonymous to an increasing degree of hydration.

Even though the initial conductivity of the pore solution had an impact on the electrical resistivity of the concrete, for SFRC, the storage medium only had a minor influence on the electrical resistivity. A higher initial alkalinity slightly reduced the electrical resistivity of the concrete. For future analysis, this implies the necessity to use constant storage conditions, namely, a certain conditioning of the specimens before the measurement of the electrical resistivity should be performed, but unknown conditions of concrete in structural elements are not an exclusion criterion for this test method.

As expected, a variation of the concrete also influenced the electrical resistivity, mostly due to changes in the pore structure of the concrete. The observed parameters, w/c ratio, binder content, cement type and GGBS content, all caused changes in the electrical resistivity.

As stated in several studies (e.g., [11,26,37,38,41,44,45,46,47,48]), the increase in porosity, which can be a result of a higher w/c ratio or due to an increased binder content, generally led to a reduced electrical resistivity. However, in contrast to [41,44,45,46,47,48], both parameters in this study showed interaction effects.

The same effect of reduced electrical resistivity was detected with the decreasing content of GGBS. Generally, GGBS has a finer pore structure than the concrete, and so the conductivity is reduced by its addition (e.g., [42,46,49,50,51,52,53,54,55,56,57,58,59,60]). In this study, no effect on the electrical resistivity was observed by the replacement of only small amounts of cement by GGBS, but with higher contents of GGBS, 65% of the total binder content, a significant change in electrical resistivity was measured. Due to the young concrete age of only 7 days, it can be assumed that the GGBS, which is a latent hydraulic binder material, only had a minor impact on the initial hydration process and hence on the resistivity of the concrete especially at replacements of 35%. Nevertheless, a higher content of GGBS apparently caused a finer pore structure, visible by the increased electrical resistivity.

For different cement types, in this case, the variation of the grinding fineness for cements with almost identical clinker composition, it was assumed that the finest material leads to the finest pore structure and thus to the lowest conductivity. Comparing the two cements with lower strength classes, the observations fit our expectations, while the highest cement fineness resulted in a decrease in electrical resistivity.

In addition to the single parameter changes, multiple simultaneous changes in the concrete composition were also investigated, and based on the investigated number of samples, the conclusion must be drawn that interaction effects do not enable the superposition of the effects of single parameter changes. Figure A1, Appendix A, shows the interaction plot of all the varied parameters. While a parallel shift of the single curves of each parameter combination would represent the independence of these parameters, no group of curves showing this behaviour is visible. On the contrary, all parameter combinations show intersections between the single curves.

Since the most influencing factor of the conductivity of the concrete, in the literature as well as in several experiments (e.g., [26,37,38]), was determined to be the porosity of the concrete, with the exception of the saturation of the pores, which is stated to be preferably high, simultaneous changes in parameters must be considered for the measurement of electrical resistivity. Thus, it is not possible to generate a database of concretes and interpolate, respectively, extrapolate the electrical resistivity of nonincluded compositions in a trivial manner; however, it is necessary to identify the composition of the concrete and, ideally, the electrical resistivity of the plain concrete if the fibre content is determined by the use of electrical resistivity measurements.

## 5. Conclusions

This study showed that the determination of the electrical resistivity of SFRC is a suitable test method for the monitoring of fibre content, e.g., in precast factories or for new construction buildings where the electrical resistivity of the plain concrete composition can be characterised. The large difference between the conductivity of steel as a metallic conductor and concrete as an ionic conductor led to significant influences of the fibre content on the electrical resistivity, which resulted in a 70% lower electrical resistivity of SFRC, 80 kg/m^3^, in comparison to plain concrete. Nevertheless, since there are also influences from the concrete composition, such as on the w/c ratio and binder content, the application field of this test method requires previous calibration on concrete mix designs without the addition of fibres. Otherwise, the effects of the concrete composition, which can influence the electrical resistivity by approximately 15% to 25%, can induce significant errors in the analysis of the fibre content. Investigations of existing structures in the case of repair or maintenance work can only lead to sufficient approximations for the fibre content if either the composition or electrical resistivity of the plain concrete are well known, e.g., with the help of an extensive database or the characterisation of plain concrete without fibres. Both possible methods of extending the test setup to enable its use for the characterisation of existing structures and additionally in the evaluation of fibre orientation are the subject our future studies.

## Figures and Tables

**Table 1:** Characterisation of the cements and supplementary cementitious materials.

Binder	Density in g/cm^3^	Specific Surface in cm^2^/g
CEM I 32.5 R	3.15	3000
CEM I 42.5 R	3.14	3670
CEM I 52.5 R	3.15	4320
GGBS	2.91	4240

**Table 2:** Parameters of the used fibres.

Parameter	Unit	Fibre 1	Fibre 2	Fibre 3
Fibre type	-	Macrofibre	Macrofibre	Microfibre
Material	-	steel wire	steel wire	steel wire
Coating	-	-	-	brass
Length	mm	60	35	8
Diameter		1	0.75	0.175
l/d ratio	-	60	46.7	45.7
Fibre quantity	pieces/kg	2600	8200	662,000

**Table 3:** Concrete mix design of the basic concrete.

Parameter	Unit	32-60-300-00
CEM I 32.5 R	kg/m^3^	300.0
GGBS		-
Water		180.0
Aggregates		1849.5
Water/cement ratio	-	0.60
Grain size distribution		A/B16
Steel fibre type		Macrofibre 60 mm
Steel fibre content	kg/m^3^	0 to 80

**Table 4:** Statistical concrete matrix with combined variations.

Concrete Designation	Cement Type	w/c Ratio	Binder Content in kg/m^3^	Amount of GGBS in %
32-55-270-35	CEM I 32.5 R	0.55	270	35
32-60-300-00 *	CEM I 32.5 R	0.60	300	0
32-65-330-65	CEM I 32.5 R	0.65	330	65
42-55-330-00	CEM I 42.5 R	0.55	330	0
42-60-270-65	CEM I 42.5 R	0.60	270	65
42-65-300-35	CEM I 42.5 R	0.65	300	35
52-55-300-65	CEM I 52.5 R	0.55	300	65
52-60-330-35	CEM I 52.5 R	0.60	330	35
52-65-270-00	CEM I 52.5 R	0.65	270	0

* Basic concrete mix design.

**Table 5:** Initial composition of synthetical pore solution.

Component	Unit	Value
KOH	mmol/L	170
NaOH		17
Ca(OH)_2_		23

**Table 6:** Effect of the single parameters on the electrical resistivity of SFRC.

Parameter	Minimum Electrical Resistivity in Ωm	Maximum Electrical Resistivity in Ωm	Percentage Influence of the Parameter in %
Frequency	22.7	24.5	7.3
Fibre content	13.8	43.5	68.3
w/c ratio	21.2	24.5	13.5
Binder content	20.0	26.2	23.7
Cement type	19.9	25.9	23.2
GGBS content	19.8	27.0	26.7
Fibre type	20.6	22.7	11.9

## Data Availability

The data presented in this study are available in Appendix A.

## Appendix A

**Table A1:** Concrete mix designs for the variation of the water/cement ratio.

Parameter	Unit	32-55-300-00	32-65-300-00
CEM I 32.5 R	kg/m^3^	300.0	300.0
GGBS		-	-
Water		165.0	195.0
Aggregates		1888.9	1810.0
Water/cement ratio	-	0.55	0.65
Grain size distribution		A/B16	A/B16
Steel fibre type		Macrofibre 60 mm	Macrofibre 60 mm
Steel fibre content	kg/m^3^	0 to 80	0 to 80

**Table A2:** Concrete mix designs for the variation of the binder content.

Parameter	Unit	32-60-270-00	32-60-330-00
CEM I 32.5 R	kg/m^3^	270.0	330.0
GGBS		-	-
Water		180.0	180.0
Aggregates		1922.3	1776.7
Water/cement ratio	-	0.60	0.60
Grain size distribution		A/B16	A/B16
Steel fibre type		Macrofibre 60 mm	Macrofibre 60 mm
Steel fibre content	kg/m^3^	0 to 80	0 to 80

**Table A3:** Concrete mix designs for the variation of the cement type.

Parameter	Unit	42-60-300-00	52-60-300-00
CEM I 42.5 R	kg/m^3^	300.0	-
CEM I 52.5 R		-	300.0
GGBS		-	-
Water		180.0	180.0
Aggregates		1849.5	1849.5
Water/cement ratio	-	0.60	0.60
Grain size distribution		A/B16	A/B16
Steel fibre type		Macrofibre 60 mm	Macrofibre 60 mm
Steel fibre content	kg/m^3^	0 to 80	0 to 80

**Table A4:** Concrete mix designs for the variation of the content of GGBS.

Parameter	Unit	32-60-300-35	32-60-300-65
CEM I 32.5 R	kg/m^3^	195.0	105.0
GGBS		105.0	195.0
Water		180.0	180.0
Aggregates		1843.7	1838.7
Water/cement ratio	-	0.60	0.60
Grain size distribution		A/B16	A/B16
Steel fibre type		Macrofibre 60 mm	Macrofibre 60 mm
Steel fibre content	kg/m^3^	0 to 80	0 to 80

**Table A5:** Concrete mix designs for the variation of the steel fibre type.

Parameter	Unit	32-60-300-00-F2	32-60-300-00-F3
CEM I 32.5 R	kg/m^3^	300.0	300.0
GGBS		-	-
Water		180.0	180.0
Aggregates		1849.5	1849.5
Water/cement ratio	-	0.60	0.60
Grain size distribution		A/B16	A/B16
Steel fibre type		Macrofibre 35 mm	Microfibre 8 mm
Steel fibre content	kg/m^3^	0 to 80	0 to 80

**Table A6:** Concrete mix designs for the multiple parameter variation—part 1.

Parameter	Unit	32-55-270-35	32-65-330-65
CEM I 32.5 R	kg/m^3^	175.5	115.5
CEM I 42.5 R		-	-
CEM I 52.5 R		-	-
GGBS		94.5	214.5
Water		148.5	214.5
Aggregates		1952.5	1721.4
Water/cement ratio	-	0.55	0.65
Grain size distribution		A/B16	A/B16
Steel fibre type		Macrofibre 60 mm	Macrofibre 60 mm
Steel fibre content	kg/m^3^	0 to 80	0 to 80

**Table A7:** Concrete mix designs for the multiple parameter variation—part 2.

Parameter	Unit	42-55-330-00	42-60-270-65
CEM I 32.5 R	kg/m^3^	-	-
CEM I 42.5 R		330.0	94.5
CEM I 52.5 R		-	-
GGBS		-	175.5
Water		181.5	162.0
Aggregates		1820.1	1912.6
Water/cement ratio	-	0.55	0.60
Grain size distribution		A/B16	A/B16
Steel fibre type		Macrofibre 60 mm	Macrofibre 60 mm
Steel fibre content	kg/m^3^	0 to 80	0 to 80

**Table A8:** Concrete mix designs for the multiple parameter variation—part 3.

Parameter	Unit	42-65-300-35	52-55-300-65
CEM I 32.5 R	kg/m^3^	-	-
CEM I 42.5 R		195.0	-
CEM I 52.5 R		-	105.0
GGBS		105.0	195.0
Water		195.0	165.0
Aggregates		1804.2	1878.1
Water/cement ratio	-	0.65	0.55
Grain size distribution		A/B16	A/B16
Steel fibre type		Macrofibre 60 mm	Macrofibre 60 mm
Steel fibre content	kg/m^3^	0 to 80	0 to 80

**Table A9:** Concrete mix designs for the multiple parameter variation—part 4.

Parameter	Unit	52-60-330-35	52-65-270-00
CEM I 32.5 R	kg/m^3^	-	-
CEM I 42.5 R		-	-
CEM I 52.5 R		214.5	270.0
GGBS		115.5	-
Water		198.0	175.5
Aggregates		1770.3	1886.8
Water/cement ratio	-	0.60	0.65
Grain size distribution		A/B16	A/B16
Steel fibre type		Macrofibre 60 mm	Macrofibre 60 mm
Steel fibre content	kg/m^3^	0 to 80	0 to 80

**Table A10:** Results of the resistivity measurements of the basic concrete mixture (32-60-300-00).

Fibre Content in kg/m^3^	Global Electrical Resistivity in Ωm at a Frequency of				
	100 Hz	120 Hz	1 kHz	10 kHz	100 kHz
0	44.1	44.0	43.7	43.4	43.0
10	34.2	34.2	33.7	33.3	32.9
20	28.5	28.4	27.8	27.4	27.1
30	24.1	24.0	23.2	22.7	22.3
40	20.9	20.7	19.8	19.3	18.8
50	18.3	18.2	17.4	16.8	16.4
60	16.6	16.5	15.6	15.1	14.7
70	15.2	15.0	14.1	13.5	13.1
80	13.7	13.5	12.5	12.0	11.6

**Table A11:** Results of the resistivity measurements of the concrete mixture 32-55-300-00.

Fibre Content in kg/m^3^	Global Electrical Resistivity in Ωm at a Frequency of				
	100 Hz	120 Hz	1 kHz	10 kHz	100 kHz
0	50.5	50.6	50.3	49.8	49.2
10	41.1	41.1	40.6	40.2	39.6
20	33.1	32.9	32.3	31.7	31.1
30	27.2	27.0	26.3	25.6	25.1
40	25.6	25.4	24.7	24.1	23.6
50	23.4	23.2	22.4	21.7	21.1
60	20.9	20.7	19.7	19.0	18.3
70	18.1	17.9	16.9	16.1	15.5
80	17.7	17.5	16.7	16.0	15.4

**Table A12:** Results of the resistivity measurements of the concrete mixture 32-65-300-00.

Fibre Content in kg/m^3^	Global Electrical Resistivity in Ωm at a Frequency of				
	100 Hz	120 Hz	1 kHz	10 kHz	100 kHz
0	40.5	40.6	40.3	40.0	39.6
10	32.2	32.1	31.9	31.5	31.1
20	26.7	26.6	26.0	25.5	25.0
30	24.9	24.7	24.1	23.4	23.0
40	20.8	20.7	20.1	19.5	18.9
50	18.6	18.4	17.7	17.1	16.7
60	17.2	17.0	16.3	15.4	14.8
70	17.1	16.9	16.1	15.2	14.9
80	16.3	16.1	15.3	14.6	14.2

**Table A13:** Results of the resistivity measurements of the concrete mixture 32-60-270-00.

Fibre Content in kg/m^3^	Global Electrical Resistivity in Ωm at a Frequency of				
	100 Hz	120 Hz	1 kHz	10 kHz	100 kHz
0	50.2	50.1	49.9	49.5	48.8
10	34.2	34.0	33.7	33.3	32.7
20	28.8	28.6	28.1	27.5	27.0
30	26.0	25.8	25.2	24.6	24.0
40	22.5	22.3	21.8	21.0	20.4
50	20.5	20.3	19.7	18.8	18.2
60	19.3	19.1	18.3	17.4	16.8
70	18.1	17.8	17.4	16.7	15.9
80	15.7	15.4	14.9	14.1	13.6

**Table A14:** Results of the resistivity measurements of the concrete mixture 32-60-330-00.

Fibre Content in kg/m^3^	Global Electrical Resistivity in Ωm at a Frequency of				
	100 Hz	120 Hz	1 kHz	10 kHz	100 kHz
0	36.7	36.7	36.3	36.2	35.9
10	27.7	27.7	27.1	27.0	26.7
20	21.8	21.8	21.1	20.8	20.5
30	19.9	19.7	19.0	18.6	18.3
40	16.5	16.4	15.7	15.2	14.9
50	14.8	14.7	13.9	13.4	13.1
60	13.7	13.5	12.6	12.1	11.8
70	12.2	12.1	11.2	10.6	10.3
80	11.3	11.1	10.3	9.7	9.3

**Table A15:** Results of the resistivity measurements of the concrete mixture 42-60-300-00.

Fibre Content in kg/m^3^	Global Electrical Resistivity in Ωm at a Frequency of				
	100 Hz	120 Hz	1 kHz	10 kHz	100 kHz
0	55.7	55.6	55.3	54.7	53.8
10	38.8	38.7	38.3	37.9	37.3
20	33.1	33.0	32.5	31.9	31.2
30	29.9	29.7	29.4	28.6	27.9
40	26.1	25.8	25.4	24.8	24.1
50	22.9	22.7	22.1	21.4	20.9
60	21.5	21.3	20.7	20.0	19.4
70	21.3	21.1	20.5	19.8	19.3
80	20.2	20.0	19.3	18.5	17.8

**Table A16:** Results of the resistivity measurements of the concrete mixture 52-60-300-00.

Fibre Content in kg/m^3^	Global Electrical Resistivity in Ωm at a Frequency of				
	100 Hz	120 Hz	1 kHz	10 kHz	100 kHz
0	39.6	39.5	39.5	38.9	38.4
10	35.4	35.2	35.0	34.3	33.7
20	28.8	28.6	28.0	27.2	26.5
30	27.5	27.3	26.8	26.2	25.5
40	24.4	24.2	23.6	22.7	22.0
50	21.8	21.6	21.0	20.3	19.7
60	20.7	20.5	20.0	19.1	18.5
70	19.6	19.3	18.8	18.0	17.3
80	18.8	18.6	18.1	17.3	16.7

**Table A17:** Results of the resistivity measurements of the concrete mixture 32-60-300-35.

Fibre Content in kg/m^3^	Global Electrical Resistivity in Ωm at a Frequency of				
	100 Hz	120 Hz	1 kHz	10 kHz	100 kHz
0	40.3	40.3	40.0	39.7	39.3
10	31.0	30.8	30.4	30.0	29.7
20	26.0	25.8	25.3	25.0	24.8
30	23.0	22.9	22.3	21.8	21.5
40	18.5	18.3	17.8	17.4	17.2
50	17.4	17.2	16.6	16.2	15.9
60	16.3	16.2	15.5	15.1	14.7
70	14.0	14.0	13.3	12.8	12.5
80	13.2	13.0	12.3	11.8	11.5

**Table A18:** Results of the resistivity measurements of the concrete mixture 32-60-300-65.

Fibre Content in kg/m^3^	Global Electrical Resistivity in Ωm at a Frequency of				
	100 Hz	120 Hz	1 kHz	10 kHz	100 kHz
0	54.5	54.3	53.9	53.6	52.8
10	43.3	43.2	42.6	42.1	41.6
20	36.1	35.9	35.3	34.8	34.3
30	28.6	28.5	27.8	27.4	26.9
40	25.0	24.8	23.9	23.4	22.9
50	23.5	23.3	22.4	21.9	21.4
60	20.1	20.0	19.2	18.7	18.2
70	18.9	18.8	18.1	17.7	17.2
80	16.9	16.9	16.1	15.5	15.1

**Table A19:** Results of the resistivity measurements of the concrete mixture 32-60-300-00-F2.

Fibre Content in kg/m^3^	Global Electrical Resistivity in Ωm at a Frequency of				
	100 Hz	120 Hz	1 kHz	10 kHz	100 kHz
0	43.1	43.0	42.7	42.5	42.1
10	36.4	36.4	35.8	35.4	35.0
20	31.0	30.9	30.2	29.7	29.4
30	25.8	25.7	25.0	24.5	24.0
40	22.8	22.7	21.5	20.8	20.3
50	21.1	20.9	19.7	19.0	18.6
60	19.0	18.8	17.6	16.9	16.5
70	17.5	17.2	15.9	15.2	14.7
80	16.1	15.9	14.6	13.8	13.3

**Table A20:** Results of the resistivity measurements of the concrete mixture 32-60-300-00-F3.

Fibre Content in kg/m^3^	Global Electrical Resistivity in Ωm at a Frequency of				
	100 Hz	120 Hz	1 kHz	10 kHz	100 kHz
0	44.1	44.0	43.8	43.5	43.1
10	36.4	36.1	34.9	34.4	33.9
20	30.5	30.2	28.4	27.7	27.2
30	26.1	25.6	23.6	22.6	22.1
40	22.6	22.3	20.0	19.0	18.3
50	19.9	19.5	17.2	16.1	15.4
60	17.5	17.1	14.9	13.7	13.1
70	15.8	15.5	13.2	12.0	11.4
80	14.5	14.1	11.9	10.7	10.1

**Table A21:** Results of the resistivity measurements of the concrete mixture 32-60-300-00-F1.

Fibre Content in kg/m^3^	Electrical Resistivity in Horizontal Direction in Ωm at a Frequency of				
	100 Hz	120 Hz	1 kHz	10 kHz	100 kHz
0	43.7	43.7	43.4	43.1	42.7
10	31.7	31.6	31.1	30.7	30.4
20	24.8	24.7	24.1	23.7	23.4
30	20.4	20.3	19.5	19.1	18.7
40	17.4	17.2	16.5	15.9	15.6
50	14.4	14.3	13.6	13.1	12.8
60	13.1	12.9	12.2	11.8	11.5
70	11.8	11.7	10.9	10.5	10.1
80	10.5	10.2	9.5	9.1	8.7

**Table A22:** Results of the resistivity measurements of the concrete mixture 32-60-300-00-F2.

Fibre Content in kg/m^3^	Electrical Resistivity in Horizontal Direction in Ωm at a Frequency of				
	100 Hz	120 Hz	1 kHz	10 kHz	100 kHz
0	43.3	43.3	43.0	42.7	42.3
10	34.4	34.4	33.7	33.3	33.0
20	28.2	28.1	27.3	26.8	26.5
30	22.1	21.9	21.4	21.0	20.4
40	18.9	18.7	17.6	16.9	16.5
50	17.6	17.4	16.3	15.7	15.3
60	15.3	15.1	14.1	13.4	13.1
70	14.0	13.8	12.7	12.0	11.5
80	12.5	12.4	11.3	10.7	10.3

**Table A23:** Results of the resistivity measurements of the concrete mixture 32-60-300-00-F3.

Fibre Content in kg/m^3^	Electrical Resistivity in Horizontal Direction in Ωm at a Frequency of				
	100 Hz	120 Hz	1 kHz	10 kHz	100 kHz
0	44.2	44.1	43.8	43.6	43.1
10	35.5	35.2	33.9	33.4	33.0
20	29.0	28.8	26.9	26.2	25.7
30	24.4	23.9	22.0	21.0	20.5
40	20.7	20.4	18.2	17.2	16.5
50	18.1	17.7	15.6	14.5	13.9
60	15.7	15.2	13.2	12.2	11.6
70	14.2	13.9	11.9	10.7	10.1
80	12.7	12.3	10.4	9.3	8.8

**Table A24:** Results of the resistivity measurements of the concrete mixture 32-60-300-00-F1.

Fibre Content in kg/m^3^	Electrical Resistivity in Vertical Direction in Ωm at a Frequency of				
	100 Hz	120 Hz	1 kHz	10 kHz	100 kHz
0	44.7	44.6	44.3	44.0	43.6
10	39.4	39.4	38.8	38.5	38.1
20	36.0	35.9	35.2	34.9	34.4
30	31.6	31.4	30.5	30.1	29.6
40	27.9	27.7	26.6	25.9	25.3
50	26.1	26.0	24.8	24.2	23.7
60	23.7	23.6	22.3	21.6	21.1
70	21.9	21.7	20.3	19.5	18.9
80	20.2	20.0	18.7	17.8	17.3

**Table A25:** Results of the resistivity measurements of the concrete mixture 32-60-300-00-F2.

Fibre Content in kg/m^3^	Electrical Resistivity in Vertical Direction in Ωm at a Frequency of				
	100 Hz	120 Hz	1 kHz	10 kHz	100 kHz
0	42.6	42.5	42.2	42.0	41.6
10	40.4	40.3	39.8	39.6	39.1
20	36.7	36.6	36.1	35.6	35.2
30	33.4	33.2	32.2	31.6	31.1
40	30.8	30.6	29.2	28.4	28.0
50	28.0	27.8	26.4	25.6	25.1
60	26.5	26.2	24.7	24.0	23.5
70	24.5	24.2	22.5	21.6	21.0
80	23.3	23.0	21.1	20.2	19.5

**Table A26:** Results of the resistivity measurements of the concrete mixture 32-60-300-00-F3.

Fibre Content in kg/m^3^	Electrical Resistivity in Vertical Direction in Ωm at a Frequency of				
	100 Hz	120 Hz	1 kHz	10 kHz	100 kHz
0	44.0	43.9	43.7	43.4	43.0
10	38.2	37.9	36.8	36.3	35.8
20	33.5	33.2	31.5	30.7	30.3
30	29.5	29.1	26.9	25.9	25.4
40	26.5	26.0	23.7	22.5	21.9
50	23.4	22.9	20.4	19.2	18.5
60	21.3	20.9	18.2	16.9	16.2
70	19.1	18.7	16.0	14.6	13.9
80	18.1	17.6	15.0	13.6	12.8

**Table A27:** Results of the resistivity measurements of the concrete mixture 32-55-270-35.

Fibre Content in kg/m^3^	Global Electrical Resistivity in Ωm at a Frequency of				
	100 Hz	120 Hz	1 kHz	10 kHz	100 kHz
0	48.2	48.1	47.7	47.5	47.0
10	39.4	39.4	38.9	38.5	38.1
20	31.4	31.3	30.7	30.3	29.8
30	25.5	25.3	24.7	24.3	24.0
40	21.4	21.3	20.7	20.2	19.8
50	20.1	19.9	19.3	18.8	18.4
60	17.0	16.9	16.2	15.7	15.3
70	16.2	16.1	15.4	15.0	14.6
80	15.7	15.6	14.9	14.4	14.0

**Table A28:** Results of the resistivity measurements of the concrete mixture 32-65-330-65.

Fibre Content in kg/m^3^	Global Electrical Resistivity in Ωm at a Frequency of				
	100 Hz	120 Hz	1 kHz	10 kHz	100 kHz
0	46.8	46.7	46.3	46.0	45.5
10	37.6	37.5	37.1	36.8	36.3
20	30.0	29.9	29.3	29.0	28.7
30	26.2	26.1	25.5	25.1	24.7
40	20.3	20.1	19.4	18.9	18.6
50	19.0	18.8	18.1	17.6	17.3
60	17.1	17.0	16.3	15.9	15.5
70	15.5	15.4	14.7	14.3	13.8
80	14.7	14.7	14.1	13.5	13.2

**Table A29:** Results of the resistivity measurements of the concrete mixture 42-55-330-00.

Fibre Content in kg/m^3^	Global Electrical Resistivity in Ωm at a Frequency of				
	100 Hz	120 Hz	1 kHz	10 kHz	100 kHz
0	42.0	42.0	41.7	41.5	40.9
10	33.3	33.2	32.8	32.5	32.1
20	27.3	27.2	26.7	26.3	26.0
30	22.5	22.3	21.8	21.4	21.0
40	19.7	19.6	19.0	18.7	18.4
50	18.0	17.9	17.4	17.0	16.7
60	14.9	14.8	14.1	13.7	13.4
70	14.2	14.0	13.4	12.9	12.5
80	12.7	12.6	11.9	11.4	11.1

**Table A30:** Results of the resistivity measurements of the concrete mixture 42-60-270-65.

Fibre Content in kg/m^3^	Global Electrical Resistivity in Ωm at a Frequency of				
	100 Hz	120 Hz	1 kHz	10 kHz	100 kHz
0	68.2	68.1	67.7	67.2	66.2
10	52.6	52.5	51.8	51.3	50.4
20	42.1	42.0	41.3	40.8	40.1
30	35.9	35.8	34.9	34.4	33.7
40	30.2	30.0	29.2	28.8	28.2
50	26.7	26.6	25.8	25.2	24.6
60	23.7	23.5	22.6	22.0	21.5
70	21.3	21.1	20.3	19.7	19.2
80	19.3	19.1	18.2	17.7	17.2

**Table A31:** Results of the resistivity measurements of the concrete mixture 42-65-300-35.

Fibre Content in kg/m^3^	Global Electrical Resistivity in Ωm at a Frequency of				
	100 Hz	120 Hz	1 kHz	10 kHz	100 kHz
0	33.0	32.9	32.7	32.5	32.2
10	26.3	26.3	25.9	25.6	25.3
20	21.6	21.6	21.2	20.9	20.6
30	18.6	18.5	17.9	17.6	17.3
40	16.5	16.3	15.8	15.5	15.3
50	13.7	13.6	13.0	12.7	12.5
60	12.2	12.1	11.5	11.2	10.9
70	12.2	12.1	11.4	11.4	11.0
80	10.4	10.3	9.8	9.5	9.2

**Table A32:** Results of the resistivity measurements of the concrete mixture 52-55-300-65.

Fibre Content in kg/m^3^	Global Electrical Resistivity in Ωm at a Frequency of				
	100 Hz	120 Hz	1 kHz	10 kHz	100 kHz
0	36.2	36.2	36.0	35.8	35.5
10	31.0	30.9	30.4	30.2	29.8
20	24.4	24.2	23.7	23.4	23.0
30	20.0	19.9	19.3	18.9	18.7
40	19.4	19.2	18.4	17.8	17.5
50	16.1	16.0	15.3	14.8	14.5
60	13.6	13.5	12.8	12.3	12.1
70	13.3	13.2	12.4	12.0	11.7
80	12.2	12.1	11.5	11.1	10.8

**Table A33:** Results of the resistivity measurements of the concrete mixture 52-60-330-35.

Fibre Content in kg/m^3^	Global Electrical Resistivity in Ωm at a Frequency of				
	100 Hz	120 Hz	1 kHz	10 kHz	100 kHz
0	24.5	24.4	24.3	24.2	24.0
10	20.8	20.7	20.4	20.2	19.9
20	17.5	17.4	16.9	16.6	16.4
30	15.5	15.4	14.9	14.6	14.3
40	13.8	13.6	13.0	12.7	12.5
50	12.5	12.4	11.8	11.5	11.2
60	11.3	11.2	10.6	10.2	10.0
70	10.2	10.1	9.5	9.2	8.9
80	9.1	9.0	8.4	8.0	7.8

**Table A34:** Results of the resistivity measurements of the concrete mixture 52-65-270-00.

Fibre Content in kg/m^3^	Global Electrical Resistivity in Ωm at a Frequency of				
	100 Hz	120 Hz	1 kHz	10 kHz	100 kHz
0	36.8	36.8	36.5	36.2	35.9
10	29.4	29.3	28.8	28.5	28.1
20	23.3	23.2	22.5	22.1	21.8
30	20.3	20.1	19.3	18.8	18.5
40	17.3	17.1	16.4	15.9	15.5
50	16.0	15.8	14.9	14.5	14.1
60	14.2	14.0	13.2	12.8	12.4
70	13.2	13.1	12.2	11.7	11.3
80	12.3	12.1	11.3	10.8	10.4

**Table A35:** Results of the resistivity measurements of the concrete mixture 32-60-300-00 after storage in H2O for 7 days for the investigations of possible leaching effects.

Fibre Content in kg/m^3^	Global Electrical Resistivity in Ωm at a Frequency of				
	100 Hz	120 Hz	1 kHz	10 kHz	100 kHz
0	40.0	40.0	39.7	39.5	39.1
40	20.1	19.9	19.2	18.8	18.4
80	12.7	12.6	11.9	11.5	11.1

**Table A36:** Results of the resistivity measurements of the concrete mixture 32-60-300-00 after storage in H2O for 14 days for the investigations of possible leaching effects.

Fibre Content in kg/m^3^	Global Electrical Resistivity in Ωm at a Frequency of				
	100 Hz	120 Hz	1 kHz	10 kHz	100 kHz
0	46.2	46.1	45.9	45.6	45.1
40	21.4	21.4	20.6	20.1	19.7
80	13.9	13.8	13.1	12.6	12.3

**Table A37:** Results of the resistivity measurements of the concrete mixture 32-60-300-00 after storage in H_2_O for 28 days for the investigations of possible leaching effects.

Fibre Content in kg/m^3^	Global Electrical Resistivity in Ωm at a Frequency of				
	100 Hz	120 Hz	1 kHz	10 kHz	100 kHz
0	50.5	51.4	51.3	51.0	50.5
40	23.8	23.7	22.9	22.3	21.8
80	15.3	15.2	14.4	13.9	13.5

**Table A38:** Results of the resistivity measurements of the concrete mixture 32-60-300-00 after storage in H_2_O for 56 days for the investigations of possible leaching effects.

Fibre Content in kg/m^3^	Global Electrical Resistivity in Ωm at a Frequency of				
	100 Hz	120 Hz	1 kHz	10 kHz	100 kHz
0	57.0	56.9	56.6	56.1	55.0
40	26.5	26.4	25.3	24.5	23.5
80	15.8	15.7	14.9	14.3	13.9

**Table A39:** Results of the resistivity measurements of the concrete mixture 32-60-300-00 after storage in H_2_O for 91 days for the investigations of possible leaching effects.

Fibre Content in kg/m^3^	Global Electrical Resistivity in Ωm at a Frequency of				
	100 Hz	120 Hz	1 kHz	10 kHz	100 kHz
0	63.0	63.0	62.3	61.2	59.2
40	30.1	30.0	28.5	27.0	25.5
80	16.3	16.1	15.2	14.6	14.2

**Table A40:** Results of the resistivity measurements of the concrete mixture 32-60-300-00 after storage in H_2_O for 185 days for the investigations of possible leaching effects.

Fibre Content in kg/m^3^	Global Electrical Resistivity in Ωm at a Frequency of				
	100 Hz	120 Hz	1 kHz	10 kHz	100 kHz
0	78.3	78.2	74.8	70.4	66.3
40	42.7	42.3	38.2	33.3	29.3
80	19.2	19.0	17.7	16.5	15.4

**Table A41:** Results of the resistivity measurements of the concrete mixture 32-60-300-00 after storage in Ca(OH)_2_ for 7 days for the investigations of possible leaching effects.

Fibre Content in kg/m^3^	Global Electrical Resistivity in Ωm at a Frequency of				
	100 Hz	120 Hz	1 kHz	10 kHz	100 kHz
0	39.4	39.4	39.1	38.9	38.5
40	18.9	18.8	18.2	17.7	17.4
80	12.6	12.4	11.8	11.4	11.1

**Table A42:** Results of the resistivity measurements of the concrete mixture 32-60-300-00 after storage in Ca(OH)_2_ for 14 days for the investigations of possible leaching effects.

Fibre Content in kg/m^3^	Global Electrical Resistivity in Ωm at a Frequency of				
	100 Hz	120 Hz	1 kHz	10 kHz	100 kHz
0	44.8	44.8	44.5	44.2	43.8
40	20.9	20.9	20.2	19.7	19.4
80	13.2	13.1	12.4	12.1	11.8

**Table A43:** Results of the resistivity measurements of the concrete mixture 32-60-300-00 after storage in Ca(OH)_2_ for 28 days for the investigations of possible leaching effects.

Fibre Content in kg/m^3^	Global Electrical Resistivity in Ωm at a Frequency of				
	100 Hz	120 Hz	1 kHz	10 kHz	100 kHz
0	49.6	49.6	49.3	49.0	48.4
40	22.2	22.1	21.4	20.9	20.5
80	14.3	14.2	13.5	13.0	12.7

**Table A44:** Results of the resistivity measurements of the concrete mixture 32-60-300-00 after storage in Ca(OH)_2_ for 56 days for the investigations of possible leaching effects.

Fibre Content in kg/m^3^	Global Electrical Resistivity in Ωm at a Frequency of				
	100 Hz	120 Hz	1 kHz	10 kHz	100 kHz
0	53.7	53.7	53.3	53.0	52.1
40	23.6	23.5	22.8	22.3	21.8
80	15.8	15.7	14.8	14.0	13.2

**Table A45:** Results of the resistivity measurements of the concrete mixture 32-60-300-00 after storage in Ca(OH)_2_ for 91 days for the investigations of possible leaching effects.

Fibre Content in kg/m^3^	Global Electrical Resistivity in Ωm at a Frequency of				
	100 Hz	120 Hz	1 kHz	10 kHz	100 kHz
0	59.2	59.2	58.7	57.7	56.2
40	26.1	25.9	25.1	24.5	24.1
80	18.8	18.6	17.3	15.7	14.4

**Table A46:** Results of the resistivity measurements of the concrete mixture 32-60-300-00 after storage in Ca(OH)_2_ for 185 days for the investigations of possible leaching effects.

Fibre Content in kg/m^3^	Global Electrical Resistivity in Ωm at a Frequency of				
	100 Hz	120 Hz	1 kHz	10 kHz	100 kHz
0	68.0	67.9	67.1	65.5	63.1
40	30.3	30.2	29.0	27.9	26.6
80	24.8	24.7	21.9	18.5	15.9

**Table A47:** Results of the resistivity measurements of the concrete mixture 32-60-300-00 after storage in synthetical pore solution for 7 days for the investigations of possible leaching effects.

Fibre Content in kg/m^3^	Global Electrical Resistivity in Ωm at a Frequency of				
	100 Hz	120 Hz	1 kHz	10 kHz	100 kHz
0	36.6	36.6	36.4	36.1	35.8
40	16.0	15.8	15.2	14.7	14.4
80	9.1	8.9	8.2	7.8	7.6

**Table A48:** Results of the resistivity measurements of the concrete mixture 32-60-300-00 after storage in synthetical pore solution for 14 days for the investigations of possible leaching effects.

Fibre Content in kg/m^3^	Global Electrical Resistivity in Ωm at a Frequency of				
	100 Hz	120 Hz	1 kHz	10 kHz	100 kHz
0	42.2	42.1	42.0	41.6	41.1
40	17.5	17.5	16.6	16.2	15.8
80	9.7	9.6	8.8	8.3	8.1

**Table A49:** Results of the resistivity measurements of the concrete mixture 32-60-300-00 after storage in synthetical pore solution for 28 days for the investigations of possible leaching effects.

Fibre Content in kg/m^3^	Global Electrical Resistivity in Ωm at a Frequency of				
	100 Hz	120 Hz	1 kHz	10 kHz	100 kHz
0	48.6	48.5	48.3	48.0	47.3
40	19.7	19.5	18.7	18.2	17.8
80	11.6	11.4	10.6	10.1	9.7

**Table A50:** Results of the resistivity measurements of the concrete mixture 32-60-300-00 after storage in synthetical pore solution for 56 days for the investigations of possible leaching effects.

Fibre Content in kg/m^3^	Global Electrical Resistivity in Ωm at a Frequency of				
	100 Hz	120 Hz	1 kHz	10 kHz	100 kHz
0	52.3	52.3	52.0	51.6	50.9
40	21.8	21.6	20.7	20.1	19.5
80	12.7	12.6	11.6	11.0	10.4

**Table A51:** Results of the resistivity measurements of the concrete mixture 32-60-300-00 after storage in synthetical pore solution for 91 days for the investigations of possible leaching effects.

Fibre Content in kg/m^3^	Global Electrical Resistivity in Ωm at a Frequency of				
	100 Hz	120 Hz	1 kHz	10 kHz	100 kHz
0	54.6	54.6	54.3	53.9	52.9
40	23.2	23.1	22.1	21.4	20.6
80	13.5	13.4	12.4	11.6	10.8

**Table A52:** Results of the resistivity measurements of the concrete mixture 32-60-300-00 after storage in synthetical pore solution for 185 days for the investigations of possible leaching effects.

Fibre Content in kg/m^3^	Global Electrical Resistivity in Ωm at a Frequency of				
	100 Hz	120 Hz	1 kHz	10 kHz	100 kHz
0	62.1	62.0	61.6	60.8	59.4
40	27.2	27.0	25.8	24.9	23.8
80	18.7	18.5	16.5	15.0	13.7
